# The dosimetric benefit of in‐advance respiratory training for deep inspiration breath holding is realized during daily treatment in left breast radiotherapy: A comparative retrospective study of serial surface motion tracking

**DOI:** 10.1111/1754-9485.13181

**Published:** 2021-05-01

**Authors:** Alan M Kalet, Aileen Kim, Daniel S Hippe, Simon S Lo, L Christine Fang, Juergen Meyer, Elvira V Lang, Nina A Mayr

**Affiliations:** ^1^ Department of Radiation Oncology University of Washington School of Medicine Seattle Washington USA; ^2^ Seattle Cancer Care Alliance Seattle Washington USA; ^3^ Clinical Research Division, Fred Hutchinson Cancer Research Center Seattle Washington USA; ^4^ Hypnalgesics, LLC Brookline Maryland USA; ^5^ Tumor Imaging and Heterogeneity Laboratory, University of Washington School of Medicine Seattle Washington USA; ^6^ Present address: Associates in Radiation Medicine Waldorf Maryland USA

**Keywords:** breast neoplasms, breath holding, heart/radiation effects, radiotherapy, respiratory training

## Abstract

**Introduction:**

A novel approach of *in‐advance* *preparatory* respiratory training and practice for deep inspiration breath holding (DIBH) has been shown to further reduce cardiac dose in breast cancer radiotherapy patients, enabled by deeper (extended) DIBH. Here we investigated the consistency and stability of such training‐induced extended DIBH after training completion and throughout the daily radiotherapy course.

**Methods:**

Daily chestwall motion from real‐time surface tracking transponder data was analysed in 67 left breast radiotherapy patients treated in DIBH. Twenty‐seven received preparatory DIBH training/practice (_prep_Trn) 1–2 weeks prior to CT simulation, resulting in an extended DIBH (_ext_DIBH) and reduced cardiac dose at simulation. Forty had only conventional immediate pre‐procedure DIBH instruction without _prep_Trn and without extended DIBH (non‐Trn group). Day‐to‐day variability in chestwall excursion pattern during radiotherapy was compared among the groups.

**Results:**

The average of daily maximum chestwall excursions was overall similar, 2.5 ± 0.6 mm for _prep_Trn/_ext_DIBH vs. 2.9 ± 0.8 mm for non‐Trn patients (*P* = 0.24). Chestwall excursions beyond the 3‐mm tolerance threshold were less common in the _prep_Trn/_ext_DIBH group (18.8% vs. 37.5% of all fractions within the respective groups, *P* = 0.038). Among patients with cardiopulmonary disease those with _prep_Trn/_ext_DIBH had fewer chestwall excursions beyond 3 mm (9.4% vs. 46.7%, *P* = 0.023) and smaller average maximum excursions than non‐Trn patients (2.4 ± 0.3 vs. 3.0 ± 0.6 mm, *P* = 0.047, respectively).

**Conclusion:**

Similar stability of daily DIBH among patients with and without preparatory training/practice suggests that the training‐induced extended DIBH and cardiac dose reductions were effectively sustained throughout the radiotherapy course. Training further reduced beyond‐tolerance chestwall excursions, particularly in patients with cardiopulmonary disease.

## Introduction

Cardiac toxicity has been recognized as one of the major long‐term risks associated with radiation therapy for breast cancer.^1^, ^2^, ^3^ Deep inspiration breath‐hold (DIBH) techniques, now widely practised for radiotherapy to the left breast and left chestwall, aims to maximize the topographic distance between the heart and the chestwall, thereby decreasing the radiation exposure of the heart from the beams that treat the target volume.^4^, ^5^, ^6^, ^7^, ^8^, ^9^, ^10^ The success of DIBH in lowering cardiac dose compared to treatment in free‐breathing mode has been well demonstrated in multiple dosimetric studies.^4^, ^10^, ^11^, ^12^

For the DIBH‐induced heart displacement from the radiation target to have clinical impact in breast cancer patients, maximal performance of each DIBH by the patient *every day during the radiation therapy course* is required. Thus the success of DIBH critically depends on the patient’s own ability to first achieve a deep inspiration through complex coordination of thoraco‐abdominal muscles, *and* then consistently maintain the DIBH daily for sufficient time while the radiation beams are delivered.

Physical and cognitive unpreparedness for these strenuous on‐demand and stringently timed physical manoeuvres can hamper the smooth and consistent performance of DIBH.^13^ Patient performance and the quality of DIBH can be variable and unpredictable among individual breast cancer patients. Similarly, patient instruction and preparation for DIBH has been highly variable, but occurs on the procedure day, shortly before the CT simulation.^9^, ^14^, ^15^, ^16^

While it has been recognized that a learning curve exists for patients to acquire the complex physical and cognitive skills to perform DIBH manoeuvres well,^17^ only very few studies have explored alternative preparatory‐training approaches to boost patients’ DIBH performance and tolerance.^13^, ^18^, ^19^, ^20^, ^21^ Logistically the timing of respiratory training can be expanded farther into the pre‐radiation therapy phase, enabling *earlier*, preparatory and thereby more intensified DIBH training regimens that provide longer time for patients to practice and improve their DIBH skills. Results of these very few studies show promise that a strategy of *early* preparatory DIBH training and practice, implemented well in advance of the first DIBH performance at the simulation procedure, can measurably decrease procedure time spent in CT simulation,^13^ lengthen the sustained DIBH^19^, ^20^ and broaden the range of breath‐hold manoeuvres performed.^21^

Among these investigations, our previous comparison study was the first to demonstrate that cardiac dose can be further reduced by in‐advance preparatory DIBH training and practice.^18^ Patients who received preparatory DIBH coaching, training and home practice for 1–2 weeks before the CT simulation, had significantly lower cardiac dose (max dose: 13.1 vs. 19.5 Gy, *P* = 0.004) than those with conventional immediate pre‐procedure instruction, based on dosimetry from CT simulation imaging. This suggests that preparatory training conditioned patients to perform a deeper, that is extended DIBH (“stretch performance”). This extended DIBH is characterized by reduced cardiac dose (compared to a conventional DIBH), likely achieved through more effective displacement of the heart from the target volume.

However, none of the early training studies^13^, ^18^, ^19^, ^20^, ^21^ evaluated whether the training‐induced DIBH “stretch performance” was maintained *during the entire treatment course*. This is an important question to answer because, if DIBH performance erodes after the training regimen concludes, during the simulation–treatment start interval and/or during daily therapy, the gains from the improved DIBH would be reduced or lost.

The purpose of this study was to determine whether the early preparatory training/practice induced “stretch performance” of an extended DIBH can be maintained after the completion of the training period and throughout the daily radiation therapy course in breast cancer patients. Specifically, we comparatively evaluated the consistency and stability of daily DIBH and chestwall excursion patterns in preparatory‐trained patients with extended DIBH, compared to patients without preparatory training and without extended DIBH.

## Methods

The records of 67 consecutive women with left breast cancer who underwent tangential radiotherapy with DIBH technique from 1/2/2015 to 31/12/2016 at our institution were analysed. This retrospective review was approved by the Institutional Review Board.

underwent tangential radiotherapy

### Patient population

The study cohort consisted of 27 patients who received in‐advance preparatory DIBH coaching, training and practice instruction by one radiation oncologist, who practised preparatory training, at least 1 week before CT simulation (_prep_Trn/_ext_DIBH group) while the control cohort consisted of 40 non‐coached patients treated in the same time period under another radiation oncologist’s care (non‐Trn group). Training was offered to all patients of the radiation oncologist, who practised DIBH training. Patients unwilling to undergo DIBH or training were excluded. Mean age among the _prep_Trn/_ext_DIBH vs. non‐Trn groups was 55.7 (±12.4) vs. 52.3 (±12.2) years (*P* = 0.30). Mean BMI was 30.1 (±7.8) vs. 26.2 (±4.8) kg/m^2^ (*P* = 0.034), respectively. Four _prep_Trn/_ext_DIBH and 7 non‐Trn patients had history of cardiac disease (including coronary atherosclerosis/history of myocardial infarction, cardiomyopathy, heart failure, valvular disease or conduction abnormalities/arrhythmia), and/or pulmonary disease (including asthma, bronchitis, COPD or history of lobectomy). Breast cancer stage distribution was similar between _prep_Trn/_ext_DIBH and non‐Trn groups (*P* = 0.17). Chemotherapy was administered prior to radiation therapy in 44.4% (12/27) vs. 45.0% (19/40) patients (*P* > 0.99), respectively. The mean radiation prescription dose was similar among the groups, 4888 (±80) cGy vs. 4879 (±100) cGy (*P* = 0.67), respectively.

To keep the training intervention as consistent and comparable as possible, this study cohort is identical to our previously reported dosimetric comparison cohort,^18^ except for two patients in the non‐Trn group, who had to be excluded from the current study (one for unavailability of sufficient daily tracking data with only six treatment fractions performed; and one in whom DIBH was planned but not performed).

### DIBH coaching and training

**Fig. 1:**
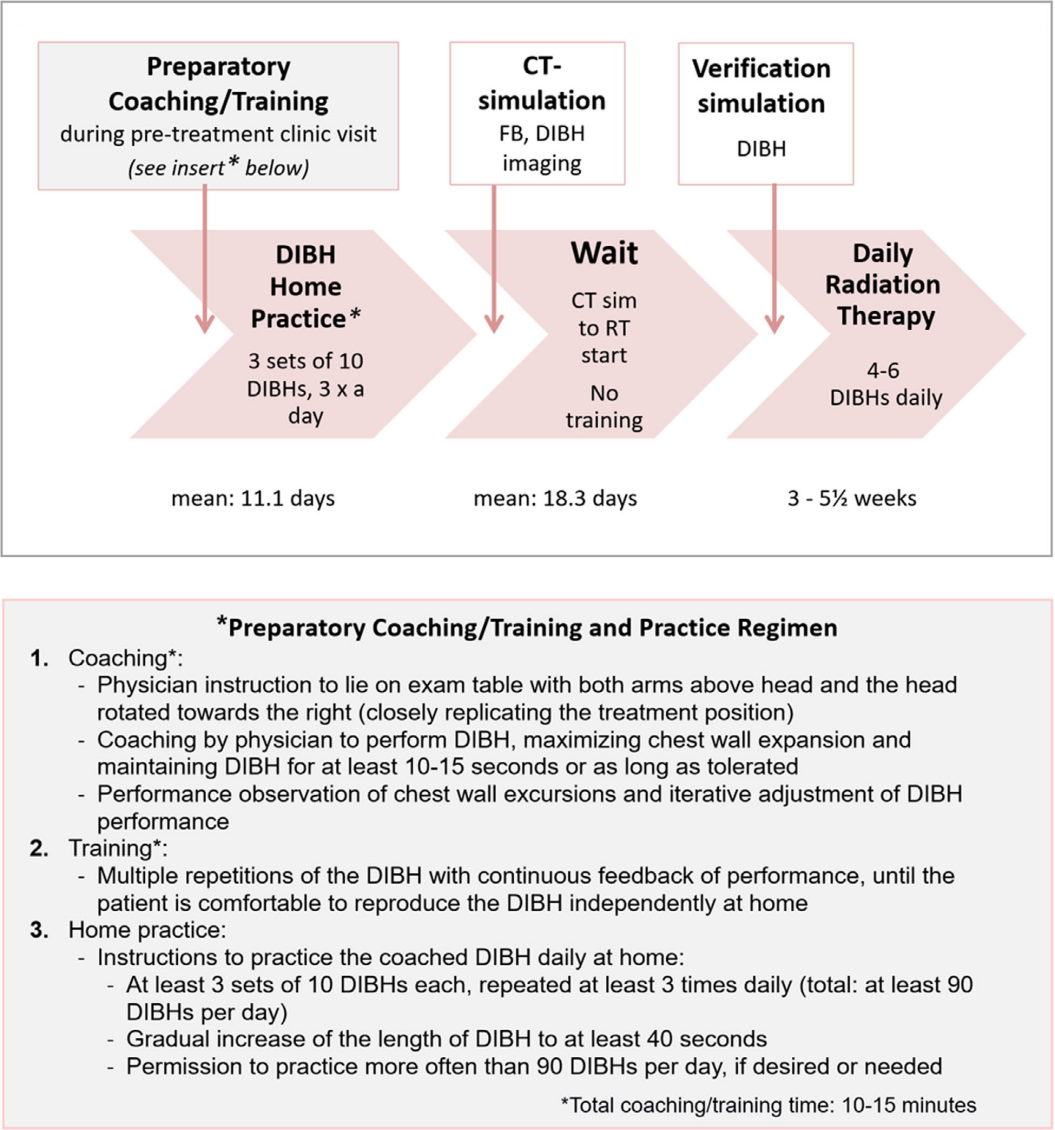
Training process and timetable of training, radiation therapy planning and treatment delivery. Boxes in top row show the interventions of coaching/training, CT simulation and verification simulation in relation to the intervening time intervals of DIBH home practice, wait time to treatment start and daily treatment course (shown in pink‐shaded arrow boxes). Corresponding duration of home practice, wait period and the treatment course are shown below pink‐shaded arrow boxes. Grey‐shaded boxes present details of coaching/training.

The DIBH training in the _prep_Trn/_ext_DIBH group occurred at least 1 week (mean: 11.1 days) before the CT simulation, as described in detail previously^18^ and illustrated in Figure 1. The non‐Trn patients under another radiation oncologist’s care received conventional immediate pre‐procedural DIBH instruction on the day of the simulation.

### Treatment planning

All patients underwent routine CT simulation on the breast board with alpha cradle immobilization. Calypso (Varian Medical Systems, Palo Alto, CA, USA) beacon markers were placed 1 cm right and 2 cm inferior of the sternal fiducial marker. An optically reflective marker was placed over the xiphoid process for visual chestwall motion tracking during simulation (RPM respiratory gating; V 1.7.5). Following a DIBH rehearsal, CTs in both free‐breathing mode and in DIBH were performed and co‐registered. Calypso beacon and isocentre positions were marked and tracked in the planning system. Contouring and planning were carried out according to routine clinical protocol with Monaco (V 5.11.01) and Xio (V 5.1.0; Elekta, Stockholm, Sweden) treatment planning systems, using 6–18 MV tangent step‐and‐shoot forward‐planned technique to provide 100% prescription dose coverage to 90–100% of the breast/chestwall volume. Calypso beacons were delineated in the treatment planning system and beacon coordinates transferred to the linear accelerator units for tracking in each treatment session.

### DIBH motion tracking

The Calypso surface beacon transponder system is a radiofrequency‐based wireless system for external real‐time motion tracking,^16^, ^22^ providing intrafraction motion tracking as an indirect surrogate for internal anatomic positioning without significantly affecting photon skin sparing in opposed tangent beam configuration.^23^, ^24^ It was found in previous studies comparing Calypso with imaging modalities such as port films and real‐time MRI that intrafractional mean motion during breath‐hold can be limited to approximately 2 mm (24, Van Heist, 2016). Patients were aligned on verification simulation and treatment days to the anteroposterior skin markers generated at simulation. The Calypso beacon was placed on the anterior chest according to the simulation‐determined position. Weekly port films were taken in DIBH. Real‐time coordinates were then zeroed out during an on‐table practice breath‐hold, setting the beacon origin (0,0,0) to the planned breath‐hold position. Treatment beams were run and manually gated by therapists when breath‐hold (as indicated by the tracking module) exceeded the predetermined tolerance limits of ±3 mm from the origin. Because the gating was performed manually, it was possible for max‐excursion values to exceed the tolerance limits before beam‐off was triggered.

### Data analysis

**Fig. 2:**
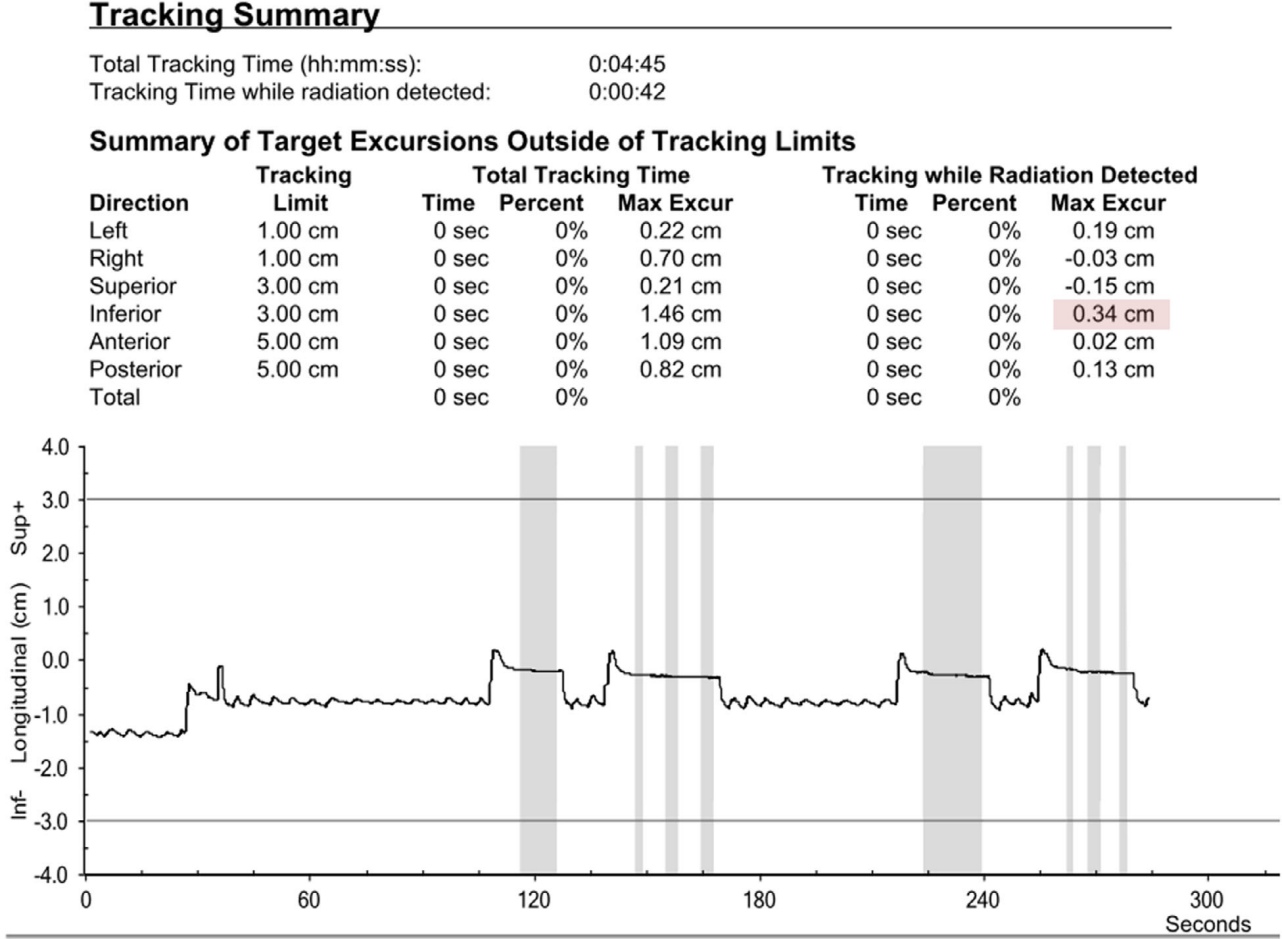
Real‐time chestwall motion tracing for DIBH. Example of a typical DIBH tracing and Tracking Summary table from the Calypso tracking system is shown. Grey‐shaded areas indicate beam‐on status. Black tracing indicates the coordinate of the beacon, parameterized by time (s). The max‐excursion is the absolute most displacement from the set‐zero‐point (i.e. target position) during beam‐on times (grey shaded). Two step‐and shoot tangential beams are presented in this patient. The table shows a maximum chestwall excursion during beam‐on time of 3.4 mm (pink‐shaded value) in the inferior direction during this treatment fraction.

Daily tracking data were extracted from patient records. Calypso generated data were processed, anonymized and analysed using R statistical programming language version 4.0.0.^25^ The chestwall *max‐excursion* was defined as the absolute value of the maximum difference between the set‐zero (i.e. target position) point and the farthest distance recorded by the Calypso beacon during beam‐on times. The max‐excursions were measured in the 3 separate directions: antero–posterior (AP), right–left (RL) and superior–inferior (SI). Figure 2 shows a graphical display of a typical breath‐hold tracing report. To make patient groups more comparable, we limited our analyses to the first 16 fractions (three weeks and one day) as a substantial proportion of our patients (15 patients) had a hypo‐fractionated radiotherapy regimen of that number of fractions.

### Statistical analysis

Continuous variables were summarized as mean ± standard deviation (SD) or median (inter‐quartile range). Categorical variables were summarized as count (percentage). Excursions were analysed in all directions (maximum of the AP, RL and SI directions) as well as in the individual directions and were analysed over the entire treatment course and within weekly treatment windows: fractions 1–5, 6–10 and 11–16. Excursions were summarized per patient by averaging max‐excursions and by calculating the percentage of fractions where max‐excursion exceeded the pre‐set 3‐mm tolerance threshold. The _prep_Trn/_ext_DIBH and non‐Trn groups were compared using the Wilcoxon rank‐sum test (continuous variables) and Fisher’s exact test (categorical variables). Longitudinal trends in average max‐excursions over time were evaluated within groups and compared between groups using linear regression with generalized estimating equations to account for repeated measurements per patient. Statistical significance was defined as a *P* < 0.05 (two‐sided) without adjustment for multiple comparisons.

### Measures to address sources of bias

Patients were identified consecutively and analysed consecutively, blinded to training status. The intake process of our multidisciplinary breast clinic assigns patients randomly at the time of *initial cancer diagnosis* (not time of radiation oncology referral) to the oncology physician team of medical, surgical and radiation oncologists. This random process, combined with our after‐the‐fact retrospective analysis likely reduces selection bias towards patient characteristics, radiation oncologist’s or referring oncologist’s patient referral preferences.

Patients from all radiation oncologists were randomly distributed to the clinic’s linear accelerators. Radiation therapists delivered daily treatment according to the same standardized DIBH procedure and were agnostic to patients’ training.

## Results

All patients were compliant in performing their DIBH for each fraction. The number of DIBH’s per fraction ranged from 2 to 6 (mean: 3.0, median: 3).

### Overall chestwall motion

**Fig. 3:**
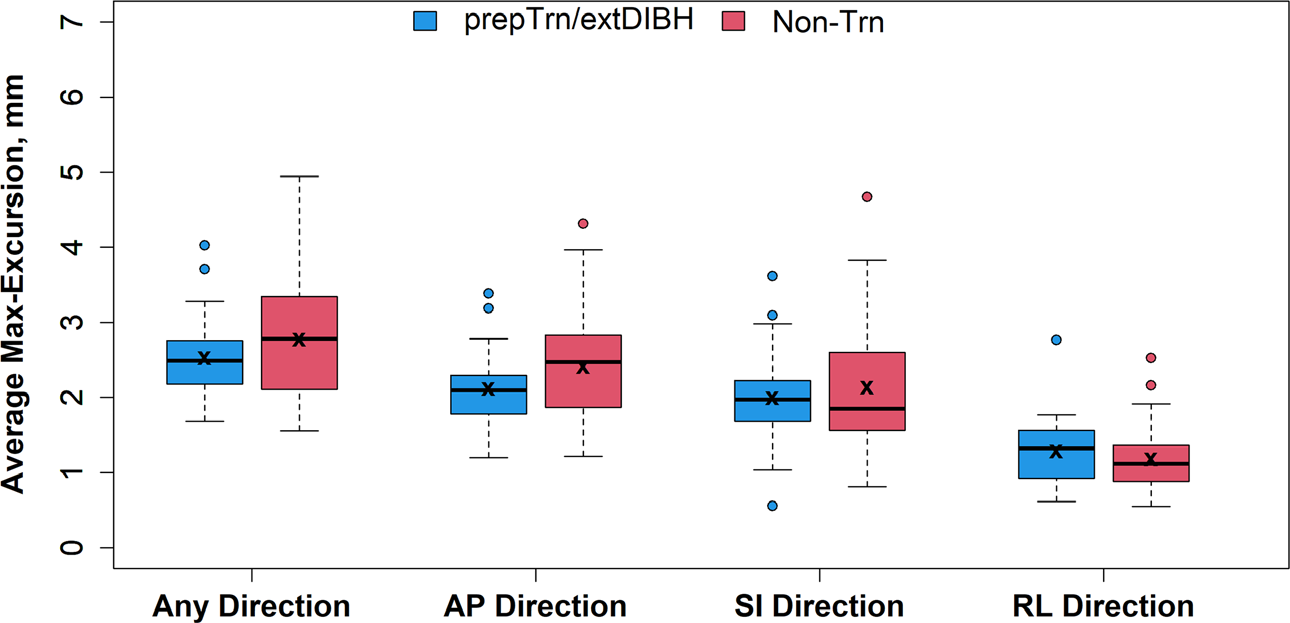
Distributions of maximal excursion of the chestwall in any direction and in the individual AP, RL and SI directions in preparatory‐trained vs. non‐trained patients. Box‐and‐whisker plots represent the median (solid line), inter‐quartile range (IQR; box) and range of the excursions in each group. The whiskers extend up to 1.5 times the IQR from the box to the smallest and largest points. Data points beyond that range are shown explicitly. The “x” indicates the mean value in each group. No differences between the preparatory‐trained (_prep_Trn/_ext_DIBH) and non‐trained (non‐Trn) groups were statistically significant (see Table 1).

The maximum chestwall excursion (max‐excursion) in any direction, averaged over the treatment course (average max‐excursion), was 2.5 ± 0.6 mm in the _prep_Trn/_ext_DIBH and 2.8 ± 0.8 mm in the non‐Trn group, showing no significant difference among the groups (*P* = 0.24; Table 1 and Fig. 3). When analysed for each direction, average max‐excursions were largest in the AP direction (mean: 2.1 ± 0.5 vs. 2.4 ± 0.7, *P* = 0.075, Table 1).

**Table 1:** Chestwall max‐excursions and excursions beyond tolerance limit in preparatory‐trained vs. non‐trained patients

	Group†		Difference		
	_prep_Trn/_ext_DIBH (*n* = 27)	Non‐Trn (*n* = 40)			
			Δ	(95% CI)‡	*P*‐value§
Average max‐excursion (in mm)					
All directions	2.5 ± 0.6	2.8 ± 0.8	−0.2	(−0.6, 0.1)	0.24
AP direction, mm	2.1 ± 0.5	2.4 ± 0.7	−0.3	(−0.6, 0.0)	0.075
SI direction, mm	2.0 ± 0.6	2.1 ± 0.8	−0.1	(−0.5, 0.2)	0.98
RL direction, mm	1.3 ± 0.4	1.2 ± 0.4	0.1	(−0.1, 0.3)	0.24
Patients with any excursion >3 mm					
All directions	22 (81.5)	35 (87.5)	−6.0	(−27.0, 15.0)	0.51
AP direction	16 (59.3)	32 (80.0)	−20.7	(−46.1, 4.7)	0.097
SI direction	16 (59.3)	28 (70.0)	−10.7	(−37.2, 15.7)	0.44
LR direction	6 (22.2)	5 (12.5)	9.7	(−12.1, 31.6)	0.33
Fractions with excursion >3 mm (in %)					
All directions	18.8 (6.2–31.2)	37.5 (12.5–56.2)	−18.8	(−36.3, 1.2)	0.038
AP direction	6.7 (0.0–18.8)	21.9 (6.9–43.8)	−15.2	(−26.1, 4.3)	0.008
SI direction	6.2 (0.0–18.8)	6.2 (0.0–26.6)	0.0	(−13.0, 13.0)	0.37
RL direction	0.0 (0.0–0.0)	0.0 (0.0–0.0)	0.0	‐	0.35

† Values are mean ± SD, no. (%), or median (inter‐quartile range).

‡ Confidence intervals are approximate.

§ Wilcoxon rank‐sum test or Fisher’s exact test comparing _prep_Trn/_ext_DIBH and non‐Trn groups.

**Fig. 4:**
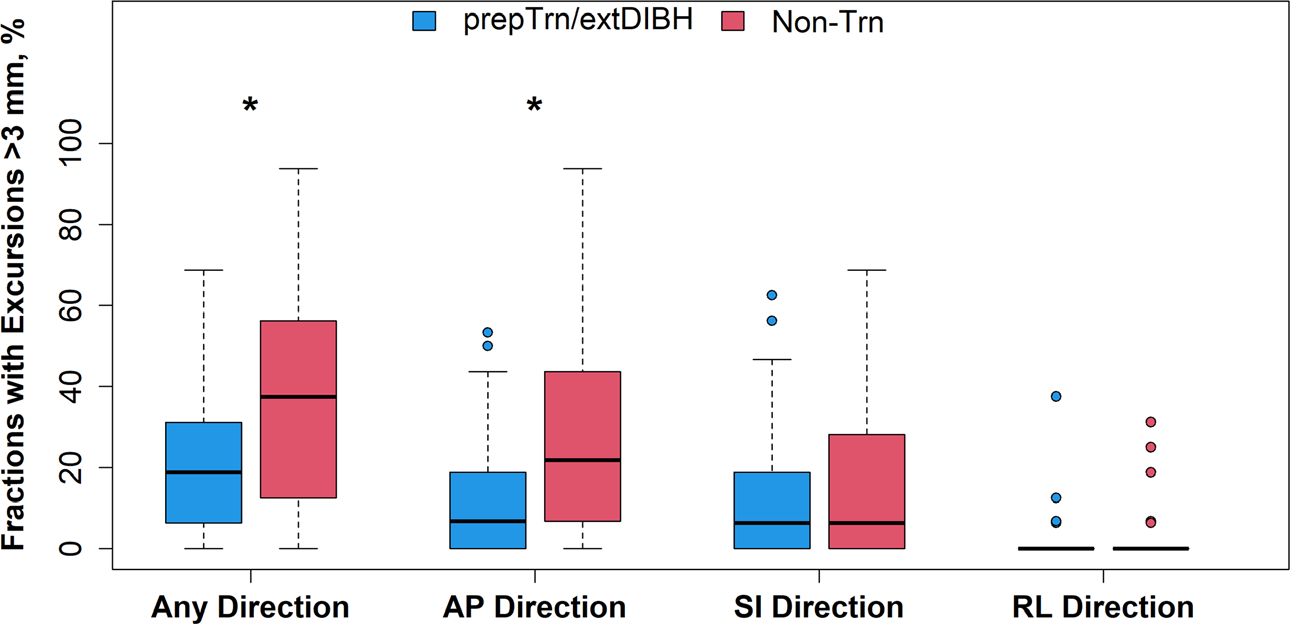
Distributions of the percentage of fractions where the chestwall excursion exceeds the 3‐mm tolerance threshold in preparatory‐trained vs. non‐trained patients. Box‐and‐whisker plots represent the median (solid line), inter‐quartile range (IQR; box) and range of the excursions in each group. The whiskers extend up to 1.5 times the IQR from the box to the smallest and largest points. Data points beyond that range are shown explicitly. The “x” indicates the mean value in each group. An asterisk indicates a statistically significant difference (*P* < 0.05) in beyond‐tolerance chestwall excursions between the preparatory‐trained (_prep_Trn/_ext_DIBH) and non‐trained (non‐Trn) group (see also Table 1).

When chestwall excursions were classified by whether they exceeded the 3‐mm tolerance threshold (in any direction), differences between the _prep_Trn/_ext_DIBH and the non‐Trn group were more apparent. Chestwall excursions beyond 3 mm in any direction were significantly less common in the _prep_Trn/_ext_DIBH group than the non‐Trn group (18.8% vs. 37.5% of all fractions, *P* = 0.038; Table 1 and Fig. 4). This difference was most pronounced in the AP direction (median: 6.7% vs. 21.9% of fractions, *P* = 0.008). We also analysed the excursions beyond 3 mm on a per patient basis. Both groups had a similar frequency of patients with one or more >3 mm excursion events (81.5% vs. 87.5%, *P* = 0.51), indicating that the significant difference in fractions with >3 mm excursion events was driven by a higher frequency of these events among patients in the non‐Trn group than among patients in the _prep_Trn/_ext_DIBH group.

### Chestwall stability over time

**Fig. 5:**
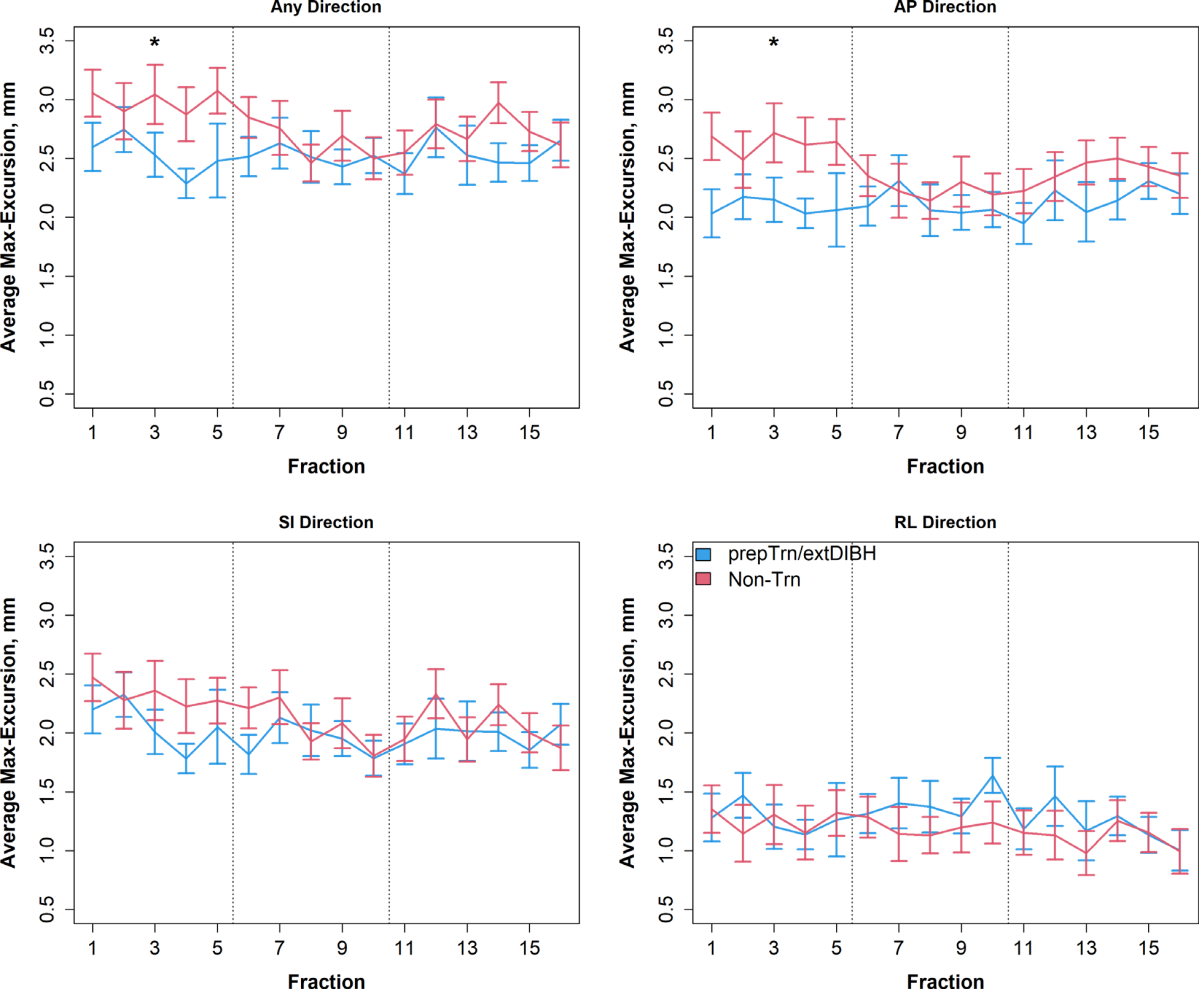
Comparison of chestwall excursion longitudinally during the radiotherapy course in each direction in preparatory‐trained vs. non‐trained patients. The maximal excursions (max‐excursions) of the chestwall were averaged at each day of treatment. Error bars indicate one standard error of the mean. The average excursions within each week (fractions 1–5, 6–10 and 11–16) were compared between _prep_Trn/_ext_DIBH and non‐Trn patient groups. The vertical dashed lines indicate the boundaries between weeks. In fraction 1–5 (first treatment week) max‐excursions in any direction and in the AP direction specifically were significantly lower in the _prep_Trn/_ext_DIBH group than the non‐Trn group.

Longitudinal analysis across the treatment course showed significantly less chestwall motion in the _prep_Trn/_ext_DIBH group compared to the non‐Trn group in the first treatment week (2.5 ± 0.7 mm vs. 3.0 ± 1.1 mm, *P* = 0.035; Table 2). Figure 5 illustrates the difference in the average max‐excursions in each direction for the _prep_Trn/_ext_DIBH and non‐Trn groups longitudinally across the treatment course. Analysis of the motion in the three directions showed the greatest difference in chestwall excursions in the AP direction (mean: 2.1 ± 0.6 mm vs. 2.6 ± 0.9 mm, *P* = 0.002; Table S1).

**Table 2:** Chestwall excursions over time in preparatory‐trained vs. non‐trained patients

	Group†		Difference		
	_prep_Trn/_ext_DIBH (*n* = 27)	Non‐Trn (*n* = 40)			
			Δ	(95% CI)‡	*P*‐value§
Average max‐excursion (in mm)					
Fractions 1–5	2.5 ± 0.7	3.0 ± 1.1	−0.5	(−0.9, −0.0)	0.035
Fractions 6–10	2.5 ± 0.7	2.6 ± 0.8	−0.1	(−0.5, 0.3)	0.75
Fractions 11–16	2.5 ± 0.6	2.7 ± 0.8	−0.2	(−0.5, 0.2)	0.47
Patients with any excursion >3 mm					
Fractions 1–5	14 (51.9)	33 (82.5)	−30.6	(−56.0, −5.3)	0.013
Fractions 6–10	18 (66.7)	24 (60.0)	6.7	(−19.8, 33.1)	0.62
Fractions 11–16	17 (63.0)	31 (77.5)	−14.5	(−40.0, 10.9)	0.27
Fractions with excursion >3 mm (in %)					
Fractions 1–5	20.0 (0.0–40.0)	40.0 (20.0–60.0)	−20.0	(−42.8, 2.8)	0.002
Fractions 6–10	20.0 (0.0–40.0)	20.0 (0.0–60.0)	0.0	(−27.7, 27.7)	0.85
Fractions 11–16	16.7 (0.0–33.3)	33.3 (16.7–66.7)	−16.7	(−39.7, 6.4)	0.035

† Values are mean ± SD, no. (%), or median (inter‐quartile range) for chestwall excursions in all directions.

‡ Confidence intervals are approximate.

§ Wilcoxon rank‐sum test or Fisher’s exact test comparing _prep_Trn/_ext_DIBH and non‐Trn groups.

In the _prep_Trn/_ext_DIBH group, there were no statistically significant trends in any direction (mean change: −3.2% to 0.9%, *P* > 0.24 for each direction). However, in the non‐Trn group there were significant reductions in average max‐excursions over time, in particular in the SI direction (mean change: −6.3% per 5‐fractions delivered, *P* = 0.003) and the RL direction (mean: change: −4.1% per 5‐fractions delivered, *P* = 0.035). Differences between these rates of chestwall excursion reductions were not statistically significant in any direction (*P* > 0.17, Table S2).

### Patients with cardiopulmonary disease

In view of the well‐known challenges in respiratory performance for patients with lung and heart disease, we separately analysed the subgroup with a history of pre‐existing cardiac and/or pulmonary disease. There were 4 such patients in the _prep_Trn/_ext_DIBH group and 7 in the non‐Trn group. While the subgroup was small, there were statistically significant differences in average max‐excursion of the chestwall in any direction (mean: 2.4 ± 0.3 mm vs. 3.0 ± 0.6 mm, *P* = 0.047). The percentage of fractions where excursions exceeded the 3‐mm tolerance threshold (median: 9.4% vs. 46.7%, *P* = 0.023) was significantly lower in the _prep_Trn/_ext_DIBH group than the non‐Trn group among the patients with cardiac and/or pulmonary disease.

## Discussion

In this study, we examined the fundamental question whether in‐advance preparatory training/practice induced gains in DIBH performance, seen at the time of simulation,^18^ can be sustained throughout the entire subsequent radiation therapy course.

### Sustaining extended DIBH throughout treatment

Our results suggest that the extended DIBH was maintained successfully during the treatment course. The overall non‐inferiority of the degree of chestwall excursions (Table 1, Fig. 3) in the _prep_Trn/_ext_DIBH group – despite their “stretch performance” of significantly deeper DIBH^18^ – suggests that the respiratory conditioning effect from the preparatory‐training regimen carried forward beyond the simulation process, across the time interval to treatment start, and throughout the treatment course.

This question was important to answer because our preparatory‐training/practice regimen was intense but *very short* in duration, ranging from 1 to 2 weeks of home practice (90 DIBH’s daily, Fig. 1), and was limited to the pre‐CT simulation phase. This is in stark contrast to the established much longer several‐month respiratory training regimens used in cardiopulmonary diseases, pre‐operative conditioning^26^, ^27^, ^28^ and in athletes.^29^ The need for timely treatment start makes such lengthy training schedules infeasible in radiation therapy. Our finding that the gains in DIBH performance from the much shorter 1–2 week pre‐simulation training regimen translated into sustained and stable daily DIBH is an important and reassuring observation for clinical management of left breast cancer patients.

This interpretation is strengthened by our study design. We intentionally examined comparative patient populations as close as possible to the comparison groups, in which we previously reported significant dosimetric cardiac dose reduction in the preparatory‐trained patients.^18^ In both studies patient management was identical among the comparison groups (preparatory training vs. conventional instruction), thereby facilitating nearly parallel comparisons among the dosimetric investigation cohort^18^ and the current study cohort where we assessed DIBH stability during actual treatment.

### Beyond‐tolerance threshold chestwall excursions

While we believe the most important result of our study is the non‐inferiority of the overall chestwall stability and DIBH consistency in patients with the preparatory training‐induced extended DIBH compared to those without the extended DIBH, our results suggest subtle evidence that preparatory training may further improve DIBH stability during treatment. In the _prep_Trn/_ext_DIBH group, the frequency of chestwall excursions beyond the 3‐mm tolerance threshold was only half of that in the non‐Trn group (18.8% vs. 37.5%, *P* = 0.038; Fig. 4).

### Chestwall stability over time

Our results also suggest that the _prep_Trn/_ext_DIBH group had a very small but statistically significant improvement in DIBH stability, shown by the smaller chestwall excursions within the first treatment week (Fig. 5), most prominently in the AP direction, where a large component of the overall chest motion is generally expected.^30^ Although the magnitude of improvement in DIBH stability is likely not clinically impactful in our population and with our current pilot training regimen, these results are intriguing. These findings may suggest that preparatory training led to a subtle improvement of DIBH stability *right away at the start* of the radiation therapy course, which was not realized by the non‐Trn group until later in therapy (Fig. 5). This implies that for non‐Trn patients the first treatment week may have served as their “training phase”, as this 1‐week time interval is strikingly similar to the 11‐day average duration of the training regimen (Fig. 1) in our _prep_Trn/_ext_DIBH patients. These preliminary observations have informed our overall preparatory‐training approach. Specifically we strive to allow an at least 1‐week training window.

### Patients with cardiopulmonary disease

Greater challenge in performing DIBH is expected for patients with cardiopulmonary disease because of the adverse impact of their pre‐existing morbidities. In contrast, we found that in patients with cardiopulmonary disease the preparatory training did not only increase the chestwall stability, but this increase was greater in the cardiopulmonary disease subgroup than that within the total patient cohort. This was shown by the larger difference in the frequency of the beyond tolerance (3‐mm) chestwall excursions between _prep_Trn/_ext_DIBH vs. non‐Trn patients among the cardiopulmonary disease group (9.4% vs. 46.7%, *P* = 0.023) compared to the difference in the total patient cohort (18.8% vs. 37.5%, *P* = 0.038, respectively).

While the cardiopulmonary subgroup was small with a total of 11 patients and this data has to be interpreted with caution, the significant training‐induced reduction of the beyond‐tolerance chestwall excursions may suggest an amplified effect of preparatory‐training cardiopulmonary disease patients, particularly when considering the *extended* DIBH that these challenging patients were able to achieve. Patients with cardiopulmonary impairment might thus particularly benefit from preparatory DIBH training, as is the case for respiratory training among patients with COPD and CHF.^27^, ^28^ Our observations show that similar training effects that have been demonstrated in cardiopulmonary/rehabilitation medicine,^26^, ^27^, ^28^ are also achievable for DIBH in radiotherapy.

### Limitations

Our hypothesis‐generating study has a relatively small patient number and should be confirmed in a larger cohort. Particularly results of the small subgroup of patients with cardiopulmonary disease should be interpreted with caution. Our retrospective design did not allow the more rigorous comparisons enabled by a randomized trial.

We cannot absolutely ascertain that patients in the _prep_Trn/_ext_DIBH group did not independently continue to practice the prescribed regimen in variable degrees after the CT simulation. Even if this was the case, our findings would continue to demonstrate that preparatory DIBH training and practice leads to an overall advantage in enhancing DIBH performance through the ability to maintain deeper DIBHs throughout the treatment course. We cannot make a clear recommendation, whether the preparatory training and practice regimen in the pre‐simulation phase is sufficient, or patients should continue practising during the time interval between simulation and treatment start. Ultimately, patients’ ability to perform the deepest possible breath hold stably and consistently – regardless of the detailed regimen used to achieve this skill – is what matters most for reducing radiation exposure of their hearts.

In conclusion, our results suggest that improvements in DIBH performance from a preparatory respiratory training and home practice regimen, performed 1–2 weeks before simulation, are sustained effectively during the subsequent daily therapy course beyond the discontinuation of training. Thus, the additional gains in heart dose reduction from preparatory DIBH training/practice are likely to be realized during actual treatment. Preparatory training may be particularly helpful in stabilizing the DIBH throughout the treatment course in patients with pre‐existing cardiopulmonary disease.

## Supporting information


**Table S1**. Chestwall excursions in different directions over time in preparatory‐trained vs. non‐trained patients.
**Table S2**. Trajectories of chestwall excursions over time within and between groups.
