# Supplementary material for: The dosimetric benefit of in‐advance respiratory training for deep inspiration breath holding is realized during daily treatment in left breast radiotherapy: A comparative retrospective study of serial surface motion tracking
Source: J Med Imaging Radiat Oncol. 2021 May 1;65(3):354–64. doi: 10.1111/1754-9485.13181 (PMC8252041; doi:10.1111/1754-9485.13181)
Supplement: Supplementary file 1 — Table S1. Chestwall excursions in different directions over time in preparatory‐trained vs. non‐trained patients. Table S2. Trajectories of chestwall excursions over time within and between groups. [file ARA-65-354-s001.docx]

**Supplemental Table 1: Chestwall excursions in different directions over time in preparatory-trained vs. non-trained patients**

|  | **Group*** | |  |  |  |  |  |
| --- | --- | --- | --- | --- | --- | --- | --- |
|  | **_prep_Trn/_ext_DIBH** | **Non-Trn** |  |  | **Difference** | | |
|  | **(n=27)** | **(n=40)** |  |  | **Δ** | **(95% CI)^†^** | **P-value^‡^** |
| **Average AP max-excursion** (in mm) |  |  |  |  |  |  |  |
| Fractions 1-5 | 2.1 ± 0.6 | 2.6 ± 0.9 |  |  | -0.5 | (-0.9, -0.2) | 0.002 |
| Fractions 6-10 | 2.1 ± 0.6 | 2.2 ± 0.7 |  |  | -0.1 | (-0.4, 0.2) | 0.57 |
| Fractions 11-16 | 2.1 ± 0.7 | 2.4 ± 0.9 |  |  | -0.2 | (-0.6, 0.1) | 0.31 |
|  |  |  |  |  |  |  |  |
| **Average SI max-excursion** (in mm) |  |  |  |  |  |  |  |
| Fractions 1-5 | 2.1 ± 0.8 | 2.3 ± 1.1 |  |  | -0.2 | (-0.7, 0.2) | 0.54 |
| Fractions 6-10 | 1.9 ± 0.7 | 2.0 ± 0.9 |  |  | -0.1 | (-0.5, 0.3) | 0.84 |
| Fractions 11-16 | 2.0 ± 0.7 | 2.1 ± 0.8 |  |  | -0.1 | (-0.4, 0.3) | 0.95 |
|  |  |  |  |  |  |  |  |
| **Average RL max-excursion** (in mm) |  |  |  |  |  |  |  |
| Fractions 1-5 | 1.3 ± 0.4 | 1.3 ± 0.5 |  |  | 0.0 | (-0.2, 0.3) | 0.58 |
| Fractions 6-10 | 1.4 ± 0.7 | 1.2 ± 0.6 |  |  | 0.2 | (-0.1, 0.5) | 0.14 |
| Fractions 11-16 | 1.2 ± 0.5 | 1.1 ± 0.4 |  |  | 0.1 | (-0.1, 0.3) | 0.49 |

Note:

* Values are mean ± SD, no. (%), or median (inter-quartile range) for chestwall excursions in all directions.

**^†^** Confidence intervals are approximate.

**^‡^** Wilcoxon rank-sum test or Fisher’s exact test comparing _prep_Trn/_ext_DIBH and non-Trn groups.

**Supplemental Table 2: Trajectories of chestwall excursions over time within and between groups.**

|  | **_prep_Trn/_ext_DIBH** | | |  | **Non-Trn** | | |  |  |
| --- | --- | --- | --- | --- | --- | --- | --- | --- | --- |
|  | **(n=27)** | | |  | **(n=40)** | | |  | **Difference** |
| **Direction** | **β*** | **(95% CI)** | **P-value†** |  | **β*** | **(95% CI)** | **P-value†** |  | **P-value‡** |
| Any | 0.4% | (-3.9, 4.8%) | 0.87 |  | -3.6% | (-7.2, 0.2%) | 0.061 |  | 0.17 |
| AP direction | 0.9% | (-4.2, 6.4%) | 0.73 |  | -3.8% | (-8.2, 0.8%) | 0.11 |  | 0.18 |
| SI direction | -1.5% | (-8.4, 5.8%) | 0.67 |  | -6.3% | (-10.2, -2.2%) | 0.003 |  | 0.25 |
| RL direction | -3.2% | (-8.4, 2.2%) | 0.24 |  | -4.1% | (-7.7, -0.3%) | 0.035 |  | 0.80 |

Note:

*β is the mean percent change in average max-excursion per 5-fraction delivered;

†Test of β = 0 within group;

‡Comparison of the changes in average max-excursion over time (β) between groups.
